# Optimization of Formulation and Processing Parameters for High-Fidelity 3D Printing of a Surimi–Flour Composite Batter

**DOI:** 10.3390/foods15142502

**Published:** 2026-07-15

**Authors:** Yaling Liu, Yaxi Peng, Xiaoxin Li, Fan Ye, Miaobin Deng, Shuai Wei, Zongyuan Han, Zefu Wang, Shucheng Liu, Yang Liu

**Affiliations:** 1 College of Food Science and Technology, Guangdong Ocean University, Zhanjiang 524088, China; 17855580217@163.com (Y.L.); pyx2746549020@163.com (Y.P.); 13924559932@163.com (X.L.); yefanfan025@163.com (F.Y.); dddxd12138@163.com (M.D.); weishuaiws@126.com (S.W.); longnv0206@163.com (Z.H.); wangzefugdou@163.com (Z.W.); lsc771017@163.com (S.L.); 2 State Key Laboratory of Marine Food Processing & Safety Control, College of Food Science and Engineering, Ocean University of China, Qingdao 266404, China; 3 Collaborative Innovation Center of Seafood Deep Processing, Dalian Polytechnic University, Dalian 116034, China

**Keywords:** 3D printing, surimi, pancake, printing conditions, batter properties

## Abstract

To enable the formulation design of 3D-printable surimi-based food systems, a surimi–flour composite batter was developed, and its printing parameters and formulation were systematically optimized using a food 3D printer, with a focus on printing accuracy, structural stability, and extrusion performance. The optimal printing parameters were identified as a flow rate of 50 mm/s, mixing time of 5 min, and syringe barrel volume of 100 mL, while the optimal formulation consisted of 25% surimi (based on flour weight), 65% water (based on total surimi–flour–water mass), and 6% butter (based on total formulation mass). Physicochemical and rheological analyses suggested that surimi promoted the transformation of bound water into immobilized water, thereby enhancing structural stability and interlayer adhesion of the printed constructs. Appropriate water addition improved hydration and extrudability, whereas butter enhanced viscoelastic properties and printing uniformity, likely through interfacial lubrication. These components collectively influenced the formation of a homogeneous gel network with pronounced shear-thinning behavior, which is essential for high-precision 3D printing. Overall, the integrated optimization of processing parameters and formulation enabled high-fidelity 3D printing of the surimi–flour composite batter, providing a formulation and processing reference for improving the printability of surimi-based composite systems in extrusion-based 3D food printing.

## 1. Introduction

Pancakes are a traditional cereal-based food produced by mixing flour with water to form a batter and then spreading it on a heated griddle for shaping and cooking. However, the traditional pancake industry has long been confronted with multiple challenges, including a single product shape, a monotonous nutritional profile and heavy reliance on empirical experience in processing [1]. These issues are mainly associated with inconsistent shaping performance and limited process standardization, which restrict the modernization of traditional pancake manufacturing [2]. 3D food printing offers a promising solution to these challenges by enabling precise, computer-controlled deposition of food materials, which allows for customized shapes, tailored nutritional profiles, and automated production [3]. Central to the successful implementation of this technology is the development of printable batter formulations with appropriate rheological properties and structural stability.

Surimi, a concentrated myofibrillar protein product derived from fish muscle through washing and refining, has attracted increasing attention in 3D food printing due to its high protein content (typically 12–18%), low fat content, and excellent gel-forming capacity. Annual global surimi production exceeds 2 million metric tons, underscoring its availability as a functional food ingredient [4]. In addition to its nutritional value, surimi exhibits excellent gel-forming ability due to its abundant myofibrillar proteins, which can form stable three-dimensional networks during processing. These properties make surimi a promising functional ingredient for extrusion-based 3D food printing. Recent studies have shown that the printability and structural stability of food inks can be improved through the construction of protein–polysaccharide composite systems, in which starches and hydrocolloids regulate rheological behavior, enhance shape retention, and improve printing fidelity [5]. Similarly, surimi–starch composites have been reported to exhibit enhanced viscoelasticity and structural stability through interactions between myofibrillar proteins and starch molecules [6,7]. These findings provide valuable insights into the development of surimi-based printable systems. Despite these advances, the application of surimi as a functional ingredient in flour-based batter systems for 3D printing remains largely unexplored, and the development of such materials remains challenging because the complex interactions among protein, starch, and lipid components may influence extrusion behavior, shape retention, and structural stability during printing [8].

Therefore, this study systematically optimized the batter formulation and processing parameters to investigate the 3D printability of a surimi–flour composite batter. We hypothesize that (i) surimi incorporation enhances the structural stability of the batter by forming a reinforced protein network; (ii) water content modulates hydration behavior and extrusion performance by regulating rheological properties; and (iii) butter improves printing fidelity through interfacial lubrication that facilitates layer deposition and shape retention. To verify these hypotheses, surimi and butter were incorporated into a flour-based batter system to construct a protein–polysaccharide–lipid composite system. The printability window and optimal processing parameters were systematically evaluated, and multi-scale characterization techniques were employed to elucidate the relationships between water distribution, viscoelastic behavior, gel network structure, and printing performance. This work provides a formulation and processing reference for improving the printability of surimi–flour composite systems in extrusion-based 3D food printing applications.

## 2. Materials and Methods

### 2.1. Materials

Frozen tilapia surimi was purchased from Honghui Aquatic Products Co., Ltd. (Beihai, China). According to the supplier, the surimi was produced from freshly harvested tilapia (*Oreochromis niloticus*) within 6 h post-harvest, processed into minced flesh, mixed with cryoprotectants (4% sucrose, 4% sorbitol, and 0.3% sodium tripolyphosphate), and frozen at −30 °C. The surimi was stored at −20 °C and used within 3 months of production. Cake flour was purchased from Zhiweichu Food Technology Co., Ltd. (Gaozhou, China). Glutinous rice flour was purchased from Gumaimeng Food Technology Co., Ltd. (Chuzhou, China). Wheat flour was purchased from Shenzhen Flour Co., Ltd. (Shenzhen, China). New Zealand-origin butter (animal-derived, from cow’s milk) was purchased from Pinyuepin Food Co., Ltd. (Shenzhen, China). According to the product specification, the butter contains ≥82% milk fat and is produced from pasteurized cow’s cream without added vegetable fats. Purified water was purchased from Wahaha Group Co., Ltd. (Hangzhou, China). Sodium chloride was purchased from Solarbio Science & Technology Co., Ltd. (Beijing, China). Iodine solution was purchased from Hewei Pharmaceutical Technology Co., Ltd. (Guangzhou, China).

### 2.2. Preparation of the Surimi–Flour Composite Batter

Thawed tilapia surimi was first homogenized for 5 min without additives using a chopper (OLK-FP01A, Olek Electric Co., Ltd., Zhongshan, China), followed by the addition of NaCl and further homogenization for another 5 min with the addition of 2.5% NaCl. Flours were sieved through a 60-mesh sieve and then mixed with surimi, butter and water to prepare the surimi–flour composite batter by homogenization using a blender (MQ 9, Braun, Melsungen, Germany) at medium speed (level 5, approximately 12,000 rpm) for 3 min, with the mixing temperature maintained at 4–8 °C using an ice-water bath. The prepared batter was stored in a refrigerator at 4 °C for subsequent use and used within 24 h.

The addition levels of all auxiliary materials were calculated according to the following methods: surimi addition = W(surimi)/W(surimi + cake flour); water addition = W(water)/W(surimi + cake flour + water); butter addition = W(butter)/W(surimi + cake flour + water + butter). The formulations of the surimi–flour composite batter were designed as follows: (1) For optimization of printing parameters: 15% surimi, 68% water and 6% butter. (2) For additive level optimization and physicochemical property analysis: different flour types (glutinous rice flour, wheat flour, cake flour); different surimi addition levels (0%, 15%, 25%, and 35%, labeled as S1, S2, S3, and S4 respectively); optimized water addition levels based on group S2 (62%, 65%, 68%, and 71%, labeled as W1, W2, W3, and W4 respectively); optimized butter addition levels based on groups S2 and W2 (0%, 3%, 6%, and 9%, labeled as O1, O2, O3, and O4 respectively). All formulations are summarized in Table 1.

### 2.3. Setting of Printing Parameters

**Figure 1:**
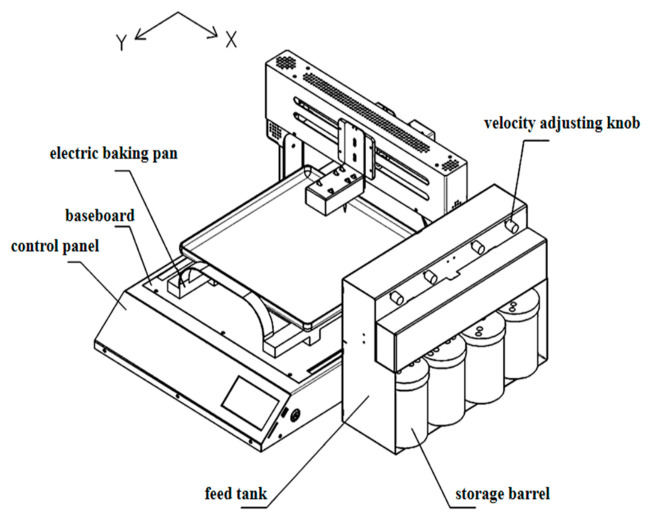
Schematic diagram of the extrusion-based pancake 3D printer and its printing process. The process involved batter preparation and loading, platform preheating and parameter setting, followed by controlled extrusion onto a heated substrate, where structure formation and stabilization were achieved via thermal induction.

Printing of the surimi–flour composite batter was performed using a pancake 3D printer (RH-J 60000, Ronghang Technology Co., Ltd., Zhengzhou, China) equipped with a printing nozzle of 1.20 mm in diameter. The working principle of the printer is shown in Figure 1. The heating platform temperature was set at 180 ± 5 °C based on preliminary experiments. The printed samples were designed as single-layer two-dimensional structures, and therefore the layer height was equal to the nozzle diameter (1.20 mm). The printer was not equipped with a pressure or force monitoring system; thus, extrusion pressure/force data were not available in this study.

The optimal printing parameters were determined by investigating the effects of batter flow rate (40, 50, 60, and 70 mm/s), stirring time (3, 5, 7, and 9 min), and syringe barrel volume (50, 75, 100, and 125 mL) on printing performance. During printing, the programmed nozzle movement speed was synchronized with the batter extrusion rate controlled by the printer software; therefore, the preset flow rate was considered the actual deposition speed in this study. For printing accuracy evaluation, three geometric models were designed: a square (50 mm × 50 mm), a circle (50 mm diameter), and a pentagram (50 mm circumscribed circle diameter). All models were printed under the optimized printing conditions.

### 2.4. Evaluation of Printing Performance

To systematically assess the optimization effect of printing parameters and ensure the representativeness of test results, a square, a circle and a pentagram were selected as the standard printing models in this study. These three shapes corresponded to key geometric features, including right-angle turning, continuous curvature and acute-angle mutation, which could comprehensively reflect the adaptability and forming fidelity of the printing system to different structures. After printing, image acquisition was conducted on the samples, and their actual projected areas were quantified using Image J software (Version 1.54d). The area fidelity (Accuracy) was calculated according to Formula (1). Finally, the arithmetic mean value of the area fidelity of the three shapes was used as the core index for the comprehensive evaluation of printing accuracy.

(1) $$Accuracy\;(\%)=(1-\frac{\left|D0-D1\right|}{D1})\times 100\%$$

In the formula, *D*1 is the area of the designed model in cm^2^; *D*0 is the area of the printed pattern in cm^2^.

### 2.5. Evaluation of Physicochemical Properties of Printing Batter

#### 2.5.1. Water-Holding Capacity (WHC)

The determination of WHC was performed according to the method described by Zhang et al. [9]. Samples were weighed and then centrifuged at 4 °C at 5000× *g* for 15 min using a high-speed centrifuge (Sigma 3-30KS, Sigma Company, Darmstadt, Germany). WHC was calculated according to Formula (2), wherein *M*0 represents the weight of the centrifuge tube, *M*1 represents the initial combined weight of sample and centrifuge tube, and *M*2 represents the weight of sample and centrifuge tube after removing the supernatant.

(2) $$WHC\;(\%)=\frac{M2-M0}{M1-M0}\times 100$$

#### 2.5.2. Low-Field Nuclear Magnetic Resonance (LF-NMR)

The method described by Monto et al. [10] was adopted for the experiment. The sample was placed into an LF-NMR tube with a diameter of 25 mm. The tube was then inserted into an LF-NMR analyzer (NMI21-060H-INMR, Newman Instrument Analysis Company, Suzhou, China), and the spin transverse relaxation time (*T*_2_) was measured at 25 ± 1 °C. The instrumental parameters were set as follows: resonance frequency at 21 MHz; spectral width at 200 KHz; radiofrequency delay time at 0.002 ms; 90° pulse width at 13.52 µs; 180° pulse width at 26 µs; waiting time at 3500 ms; echo time at 0.4 ms; number of echoes at 3500; and number of acquisitions at 16. The transverse relaxation time *T*_2_ of the samples was measured under the above conditions.

#### 2.5.3. Rheological Properties

According to the previously reported method from Yuan et al. [11], the determination was carried out using a rheometer (Haake Mars, Thermo Fisher Scientific, Waltham, MA, USA). The plate gap was set at 1 mm, the dynamic oscillation scanning frequency range was set to 0.1–10 Hz, and the composite modulus (G*) was recorded. Additionally, the shear rate range was set at 0.1–100 s^−1^, and the variation law of static apparent viscosity with shear rate was measured.

#### 2.5.4. Microscopic Characteristic

Referring to the method described by Hu et al. [12], the microscopic characteristics of the batter samples were observed by using an optical microscope (OLYMPUS-CX43, Olympus Optical Company, Tokyo, Japan) equipped with a high-performance digital camera. A drop of 0.2% (*w*/*v*) iodine–potassium iodide solution (I2:KI = 1:2, in distilled water) was added onto the surface of the sample for staining, and micrographs were obtained under a 100× objective lens.

#### 2.5.5. Confocal Laser Scanning Microscopy (CLSM)

CLSM observation was performed according to Wang et al. [13] with modifications. Briefly, approximately 1 g of freshly prepared batter was stained with three fluorescent dyes to visualize proteins, polysaccharide/starch regions, and lipids. FITC (10 μL, 2 mg/mL in ethanol) was used for protein labeling; Concanavalin A–Alexa Fluor 647 conjugate (ConA-AF647, 10 μL, 2 mg/mL in distilled water), which specifically binds to polysaccharide structures, was used for starch labeling; and Nile Red (10 μL, 2 mg/mL in ethanol), a lipid-specific probe, was used for lipid staining. After incubation in the dark for 10 min at room temperature, the stained sample was directly placed onto a glass slide for CLSM observation. Images were acquired using a confocal laser scanning microscope (LSM 880, Zeiss, Oberkochen, Germany) at 60× magnification. FITC, Nile Red, and ConA–AF647 were excited at 488, 560, and 647 nm, respectively. Fluorescence signals were collected in the green (500–550 nm), red (570–630 nm), and far-red (660–750 nm) channels, respectively. The far-red channel (ConA-AF647) was pseudo-colored blue in the merged images for better visualization.

#### 2.5.6. Particle Size

According to the previous method by Zeng et al. [14], the particle size of batter samples with different formulations was measured using a laser particle size analyzer (MS200, Malvern Panalytical, Malvern, UK). The samples were dispersed in deionized water at a ratio of 1:1000 and homogenized by stirring at 2000 rpm. Both the sample measurement duration and background measurement duration were set at 5 s. Following the measurements, we calculated the average particle size of the samples using the Mastersizer 2000 software.

#### 2.5.7. Differential Scanning Calorimetry

DSC analysis was performed using a synchronous thermal analyzer (Model DSC 3, Mettler Toledo Corp, Greifensee, Switzerland) following the method described by Yu et al. [15]. Approximately 5 mg of freshly prepared wet batter was accurately weighed into an aluminum crucible, which was then sealed with a pinhole in the lid to allow pressure release during heating; an empty aluminum crucible served as the reference. The samples were heated from 20 °C to 170 °C at a rate of 10 °C/min under a nitrogen atmosphere. DSC thermograms were analyzed using NETZSCH Proteus Thermal Analysis software (Version 9.3.1). Endothermic peaks were integrated using a manually defined linear baseline (BL) between the onset temperature (T_0_) and conclusion temperature (Tc). The software automatically normalized the integrated heat flow by the measured wet sample mass, and the enthalpy values (ΔH) are therefore reported as J g^−1^ of the total batter (wet basis).

### 2.6. Statistical Analysis

Experimental data were expressed as the mean ± standard deviation (SD) from three independent replicates (*n* = 3). For each batch of samples, all measurements were performed in triplicate. Differences at the 95% confidence level were determined via one-way analysis of variance (ANOVA) followed by Tukey’s HSD multiple comparison test, using JMP 10.0 software (SAS Institute Inc., Cary, NC, USA). Graphs were prepared using Origin 2024b (Origin Lab Inc., Northampton, MA, USA).

## 3. Results

### 3.1. Effects of Printing Parameters on Printing Fidelity

#### 3.1.1. Batter Flow Rate

**Figure 2:**
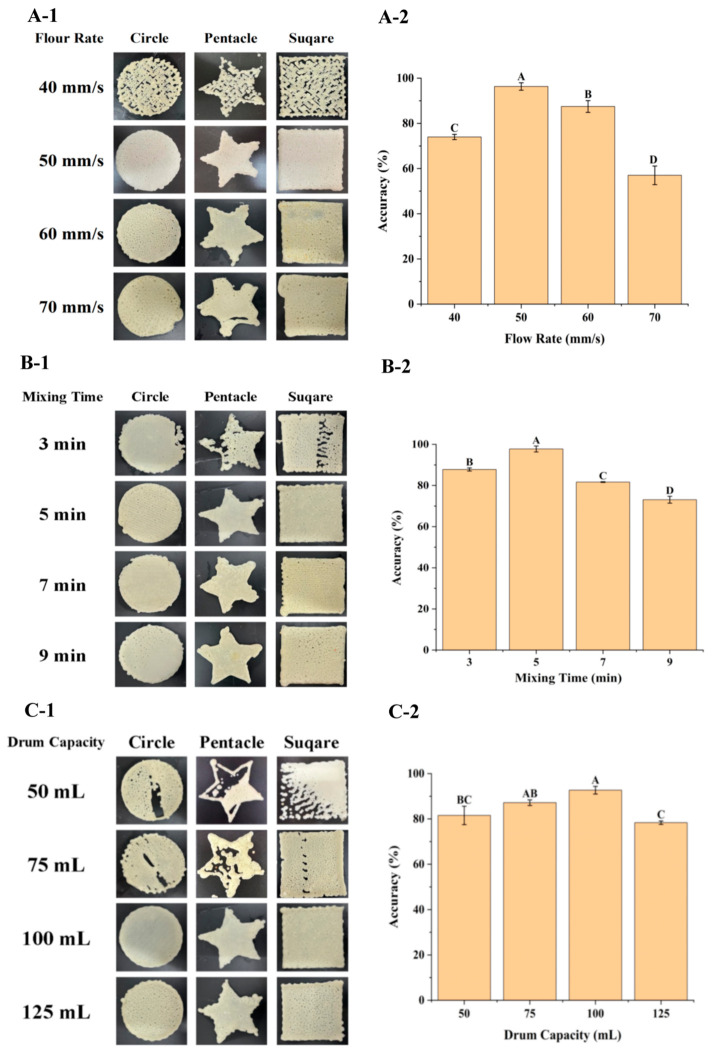
Effect of printing parameters on the printing fidelity and dimensional accuracy of surimi-flour-composite-batter. (**A-1**,**A-2**) flow rate; (**B-1**,**B-2**) mixing time; (**C-1**,**C-2**) drum capacity. For each treatment, sub-image 1 shows the representative printed patterns and sub-image 2 shows the corresponding printing accuracy. Different letters of the same parameter indicate significant differences between the average values (*p* < 0.05). Data are presented as mean ± SD (*n* = 3).

The batter flow rate represents the extrusion speed of the material through the feeding system (mm/s), and it directly influences the continuity of extrusion and the dimensional accuracy of printed structures [16]. As shown in Figure 2(A-1), obvious structural defects were observed when the flow rate was set at 40 mm/s. This phenomenon is consistent with previous findings that insufficient material supply leads to discontinuous filament deposition, reduced filament diameter, weakened interlayer bonding, and increased structural porosity [17]. As a result, the printed constructs exhibited discontinuous deposition and reduced printing fidelity. When the flow rate was increased to 50 mm/s, the printing performance was significantly improved. The printed constructs exhibited smooth and uniform surfaces without obvious structural defects, and the fidelity exceeded 98% (Figure 2(A-2)). This improvement may be attributed to a better balance between material supply and nozzle movement, allowing continuous filament deposition and stable layer-by-layer construction. However, further increasing the flow rate resulted in a gradual decline in printing accuracy. Excessive material deposition caused filament thickening, extrusion expansion, and deformation of the printed structures. This phenomenon suggests that the amount of extruded material exceeded the geometric requirements of the designed printing path, leading to material accumulation and reduced dimensional precision. Therefore, based on the printing fidelity, deposition stability, and dimensional accuracy of the printed constructs, the optimal batter flow rate was determined to be 50 mm/s.

#### 3.1.2. Mixing Time

Surimi, flour, and butter constitute a composite batter system, and their mixing uniformity directly influences rheological properties, extrusion stability, and printing performance. As shown in Figure 2(B-1), obvious local defects and insufficient interlayer adhesion were observed in the printed constructs when the mixing time was set at 3 min. This finding was consistent with research results on wheat starch systems [18], which might be attributed to the uneven dispersion of solids caused by insufficient mixing time, leading to the formation of agglomerates that easily caused nozzle clogging during extrusion and thus impaired continuous extrusion. When the mixing time was extended to 5 min, the materials reached an optimal mixing state with uniformly dispersed solid phases, extrusion continuity was significantly improved, and printing accuracy exceeded 95% (Figure 2(B-2)). However, excessive mixing time (9 min) led to material overflow and dimensional expansion, which may be attributed to over-softening of the batter structure and reduced viscosity, thereby impairing extrusion control.

#### 3.1.3. Syringe Barrel Volume

Syringe barrel volume directly affects extrusion continuity and shape retention of printed constructs. Frequent batter replenishment may affect material uniformity, which was consistent with previous findings on starch-based materials [19]. However, an excessive one-time loading would increase the material static pressure, which may affect extrusion uniformity; in addition, prolonged standing might induce component segregation, partial solidification, or alterations in the rheological properties of the bottom material. In summary, the optimal printing stability was achieved when the syringe barrel volume was set at 100 mL (Figure 2(C-1)). Under this condition, the printing process exhibited excellent continuity without the need for frequent mid-process replenishment, stable deposition was maintained, and the dimensional accuracy reached 92.67 ± 1.69% (*p* < 0.05) (Figure 2(C-2)).

### 3.2. Effects of Batter Formulation on Printability and Printing Fidelity

#### 3.2.1. Flour Type

In this study, three commonly used flours, including glutinous rice flour, wheat flour and cake flour, were compared to evaluate their effects on printing performance as the main matrix of the surimi–flour composite batter. To better interpret the observed differences in printing behavior, the intrinsic compositional characteristics of the flours, particularly protein and gluten content, were considered as key factors influencing the rheological properties and shape retention of the printing batter.

**Figure 3:**
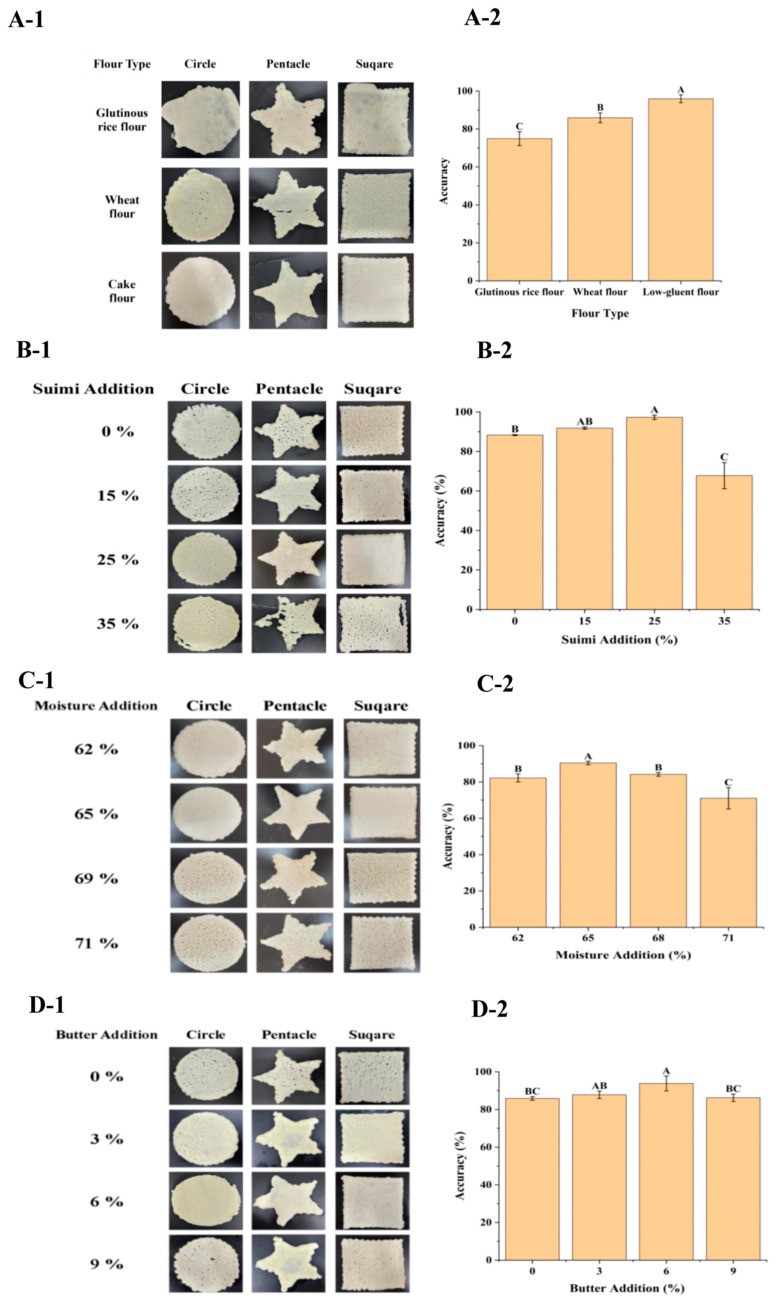
Effects of ingredient composition on the printing fidelity and dimensional accuracy of surimi–flour composite batter ((**A-1**,**A-2**) flour type; (**B-1**,**B-2**) surimi addition; (**C-1**,**C-2**) moisture addition; (**D-1**,**D-2**) butter addition). For each treatment, sub-image 1 shows the representative printed patterns and sub-image 2 shows the corresponding printing accuracy. Different letters of the same parameter indicate significant differences between the average values (*p* < 0.05). Data are presented as mean ± SD (*n* = 3).

As shown in Figure 3(A-1), the three patterns printed with the batter added with glutinous rice flour exhibited severe edge overflow and obvious deformation, with a printing accuracy of only 74.94%. This might be attributed to the lack of gluten protein, which made it difficult to form a stable supporting network, and the significant thermally induced stickiness, leading to material dragging and shape distortion [20]. Wheat flour, with a relatively high gluten protein content, could form a continuous and stable network after hydration, providing structural support for layer-by-layer deposition and maintaining shape stability. However, the strong gluten network also restricted fluidity, resulting in numerous air pores and a rough, discontinuous appearance of the printed constructs during printing [21]. In contrast, cake flour (low-gluten flour) presented excellent printing performance with a printing accuracy of 95.96%, and the printed constructs exhibited improved surface smoothness and printing fidelity.

In this study, printability was selected as the primary evaluation indicator for flour screening, as it directly reflects extrusion behavior, shape retention, and structural stability during 3D food printing. Therefore, based on printing performance, cake flour was determined as the optimal main flour for the surimi–flour composite batter.

#### 3.2.2. Surimi Addition

Although surimi exhibits favorable 3D printability [22], further investigation is required to determine whether the incorporation of surimi into flour affects the stability of the batter. As shown in Figure 3B, with the increase in surimi content, the surface roughness of the printed constructs decreased and interlayer continuity was enhanced. This was mainly attributed to the three-dimensional network structure formed between surimi proteins and starch molecules, which rendered the internal structure of the batter more compact and uniform and improved the surface flatness of the constructs [23]. However, when the surimi content was increased to 35%, the printed constructs exhibited severe defects such as incomplete patterns and large pores, which might be due to the excessively high surimi content leading to inadequate homogenization of the batter [24]. Therefore, the optimal surimi addition level in the composite batter was determined to be 25%.

#### 3.2.3. Moisture Addition

Moisture addition modulates the consistency of the printing batter and directly affects its fluidity and homogeneity. As shown in Figure 3(C-1), when the moisture addition was set at 62%, the batter failed to form sharp corners during printing and exhibited obvious defects such as material accumulation and insufficient filling, which was consistent with previous findings on sodium alginate–xanthan gum composite hydrogels [25]. When the moisture addition was 65%, the printed constructs had fewer surface bubbles, a smooth morphological appearance and high printing fidelity (Figure 3(C-2)). A further increase in moisture addition led to the formation of numerous pores in the constructs, which might be attributed to excessive water content affecting structural formation and shape retention [25]. Therefore, the optimal moisture addition for the composite batter was determined to be 65%.

#### 3.2.4. Butter Addition

Butter can improve the extrusion behavior of the printing batter by reducing interfacial friction. As shown in Figure 3D, the printing fidelity increased gradually with the increase in butter addition. This might be attributed to the fact that the incorporation of lipids reduced the interfacial friction between protein particles and provided a lubricating effect, which improved interlayer continuity and deposition uniformity and rendered the surface of printed constructs smoother and finer [26,27]. However, an excessive addition of butter resulted in numerous air pores on the surface of printed constructs, which was consistent with previous findings [28]. Therefore, the optimal butter addition for the composite batter was determined to be 6%.

### 3.3. Evaluation of Printing Fidelity and Morphological Characteristics of Printed Constructs

**Figure 4:**
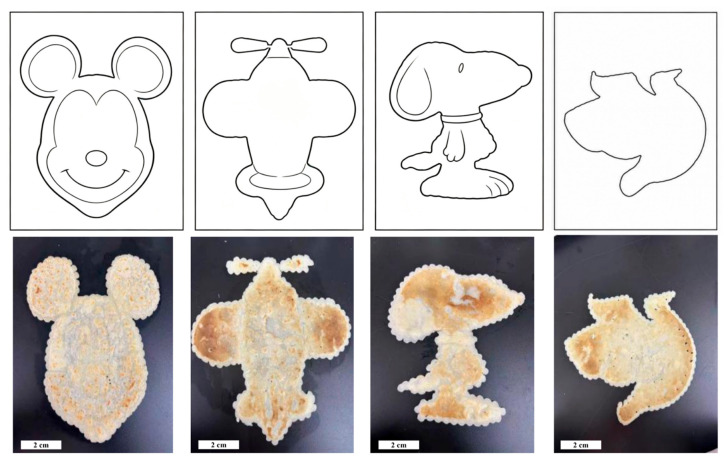
Photographs of 3D-printed surimi–flour composite batter constructs.

Using the optimized formulation (cake flour, 25% surimi, 65% water, 6% butter) and printing parameters (flow rate 50 mm/s, mixing time 5 min, syringe barrel capacity 100 mL), the surimi–flour composite batter was printed for evaluation. The printed constructs exhibited smooth surfaces, clear edge contours, and high geometric fidelity (Figure 4). The printing accuracy of the optimized formulation exceeded 95%. These results demonstrate the favorable printability and shape retention of the composite batter system, confirming the effectiveness of the optimized formulation and processing parameters for extrusion-based 3D food printing.

### 3.4. Physicochemical Characterization of Surimi–Flour Composite Batter

#### 3.4.1. Analysis of Water-Holding Capacity (WHC)

**Figure 5:**
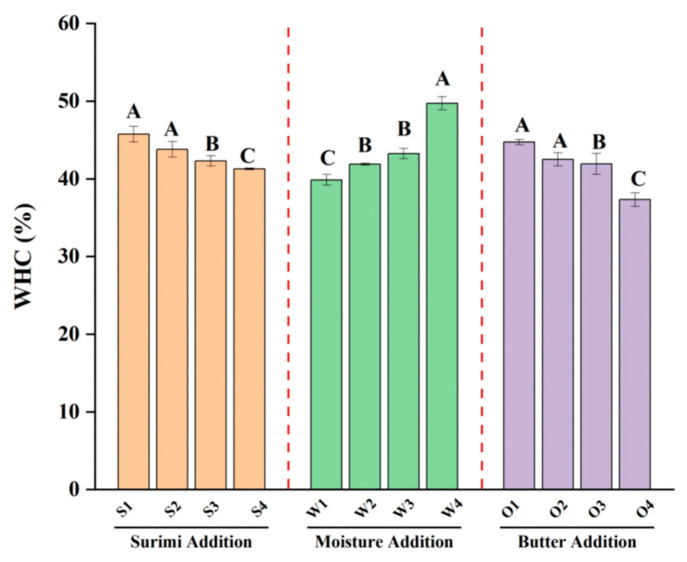
The effect of ingredient composition on the WHC of surimi–flour composite batter. Different letters of the same parameter indicate significant differences between the average values (*p* < 0.05). Data are presented as mean ± SD (*n* = 3).

Water holding capacity (WHC) reflects the water-retention ability and internal hydration state of the composite batter network [7]. The results in Figure 5 indicated a negative correlation between surimi addition and the WHC of the printing batter. It is worth noting that in pure surimi gel systems or under optimized conditions (e.g., with polysaccharide addition or cross-linking agents), surimi proteins can enhance WHC [29,30]. However, in the present flour-based composite system, a different trend was observed. This negative correlation might be associated with the competitive water absorption effect. Myofibrillar proteins and gluten proteins compete for available water molecules within the system; however, the former bind to water primarily through weak physical interactions, while gluten proteins can firmly lock in water via strong hydrogen bonding and capillary forces to form a more compact and stable hydration network [31]. Therefore, an increase in the surimi proportion essentially dilutes the fraction of the strongly bound water phase in the system, resulting in a reduction in the overall water holding capacity. This finding is specific to the flour-dominant composite system, where gluten serves as the primary water-retaining network former and surimi partially alters the dominant gluten-based hydration network, leading to reduced WHC under the present formulation conditions.

Moisture addition is also one of the key factors influencing the WHC of the surimi-based printing batter. As shown in Figure 5, within the experimental range, the WHC of the system increased with the rise in moisture addition. From the perspective of printing performance, the batter exhibited high viscosity and was in a water-deficient state when the moisture addition was 62%. Thus, moisture supplementation to the batter can drive the transition of the batter from a poorly hydrated state—characterized by water limitation and an underdeveloped network structure—to a fully hydrated state with sufficient water and a fully expanded and optimized network structure. In this process, water acts as a reactant for constructing the hydration layer, promoting the formation of a compact and homogeneous composite protein gel network in the system [32], which produces a steady improvement in water-holding capacity.

In addition, the incorporation of butter into the surimi–flour batter system led to a decrease in WHC, which was consistent with previous findings [33]. As a hydrophobic phase, butter disrupts the hydrophilic protein–starch network, forms water migration channels, and competitively weakens the hydration of components, impairing the stability of the hydration layer and converting ordered bound water into labile free water [34]. Therefore, the addition of butter to the surimi–flour composite batter results in a slight reduction in the WHC of the system.

#### 3.4.2. Analysis of Low-Field Nuclear Magnetic Resonance (LF-NMR)

**Figure 6:**
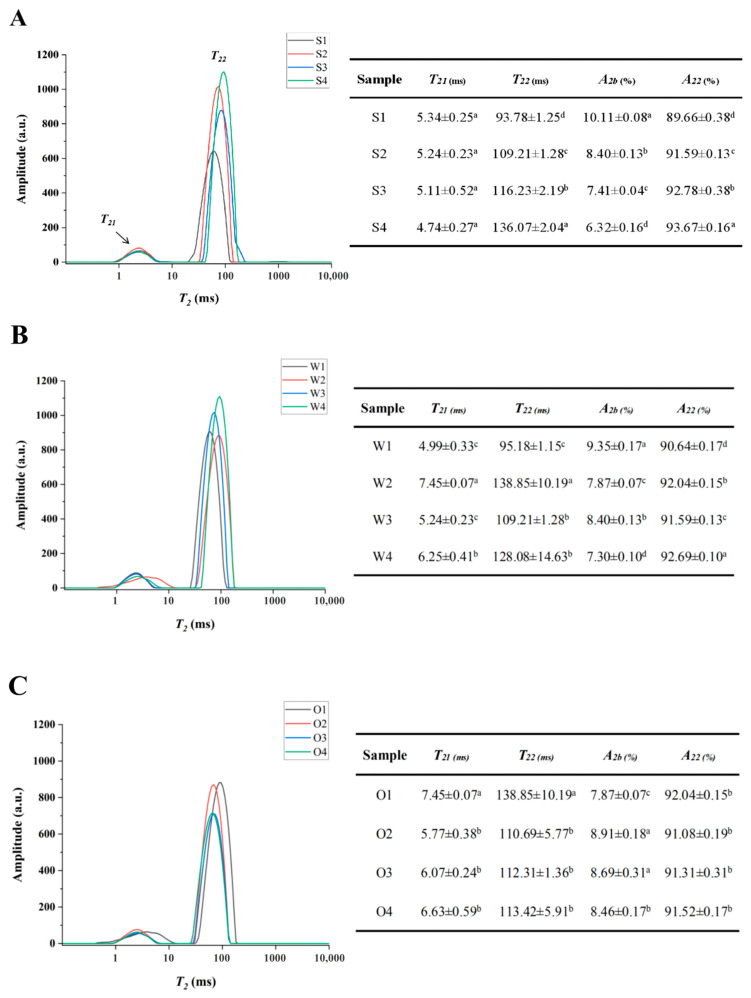
The effect of ingredient composition on water distribution in surimi–flour composite batter (**A**) surimi addition; (**B**) moisture addition; (**C**) butter addition. Different letters of the same parameter indicate significant differences between the average values (*p* < 0.05). Data are presented as mean ± SD (*n* = 3).

Low-field nuclear magnetic resonance (LF-NMR) is a non-destructive detection technique that can reflect the water distribution and migration in samples [35]. As shown in Figure 6, the relaxation curves of the surimi–flour composite batter all exhibited two peaks, namely T_21_ (0–10 ms) and T_22_ (10–200 ms). Among them, T_21_ represents the bound water tightly associated with macromolecular substances, and T_22_ denotes immobilized water trapped within the network structure.

As shown in Figure 6A, the incorporation of surimi into the flour batter caused a decrease in the relaxation time and peak area of bound water (T_21_), while the peak area of immobilized water (A_22_) increased from 89.66% to 93.67%. Concurrently, the relaxation time of immobilized water (T_22_) increased from 93.78 ms to 136.07 ms, indicating a redistribution and increased mobility within this water fraction. It should be noted that WHC measures total water retained under centrifugal force and is strongly influenced by the integrity of the gluten-based network, which is partially diluted by surimi incorporation, leading to reduced WHC. In contrast, LF-NMR reflects water distribution under equilibrium conditions, revealing that a higher proportion of water is immobilized within the newly formed surimi–starch–protein network. Therefore, although WHC decreases, LF-NMR indicates an increase in the immobilized water fraction, reflecting a redistribution of water within a reorganized gel matrix.

Moisture addition is also a key factor regulating the water distribution state of the surimi–flour batter composite system. As shown in Figure 6B, with the increase in moisture addition, the relaxation times of both bound water and immobilized water exhibited a non-linear trend of first increasing and then decreasing. This phenomenon indicates that water plays a dual role as both a plasticizer and a structure-forming medium in the system [36]. An appropriate moisture level promotes protein unfolding and the formation of a continuous gel network upon heating [37], thereby ensuring suitable rheological behavior for 3D printing.

A small amount of butter addition caused minor changes in the water distribution of the printing batter, mainly reflected in a reduction in the relaxation time of immobilized water (Figure 6C). The incorporation of butter fat globules induces rearrangement of water molecules at hydrophobic interfaces, forming interfacial water layers. This process drives a redistribution of water from strongly protein-associated states to more weakly associated interfacial water. Therefore, butter addition reduces the overall water-binding capacity of the system. Based on these changes, the water distribution state inevitably influences the rheological properties of the composite system.

#### 3.4.3. Analysis of Rheological Properties

An ideal 3D printing material should exhibit significant shear-thinning behavior and adequate viscoelastic strength, which can be evaluated by rheological parameters. Viscosity determines the extrudability of materials, while the overall viscoelastic strength of the system governs structural stability and shape retention during and after deposition [38]. In this study, the composite modulus (G*) was used to reflect the overall viscoelastic response of the printing batter under deformation. Although G* does not separately distinguish elastic and viscous contributions, it provides a reliable measure of the structural resistance relevant to extrusion and printing performance.

**Figure 7:**
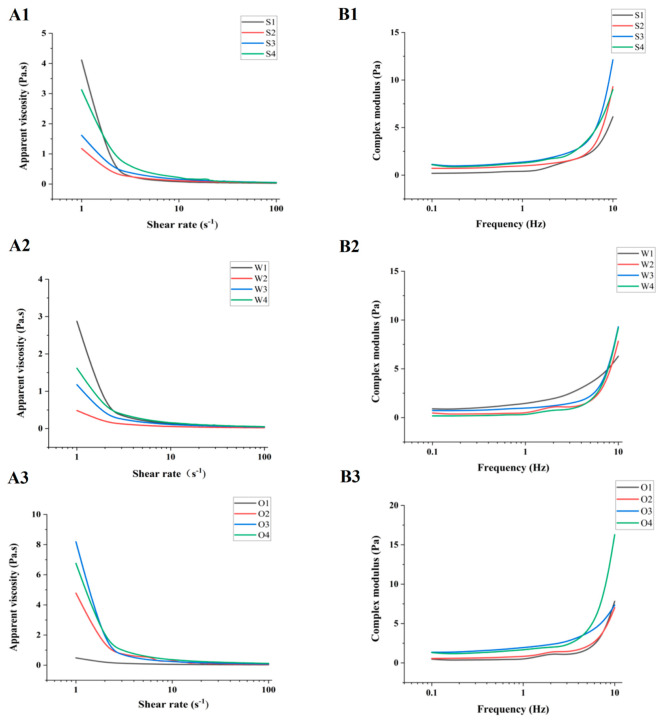
The effect of ingredient composition on rheological characteristics of surimi–flour composite batter. (**A1**–**A3**) The change in apparent viscosity with shear rate; among them, (**A1**) surimi Addition; (**A2**) moisture addition; (**A3**) butter addition.(**B1**–**B3**) Variation in composite modulus with frequency; Among them, (**B1**) surimi Addition; (**B2**) moisture addition; (**B3**) butter addition. Data are presented as mean ± SD (*n* = 3).

As shown in Figure 7, the pure flour batter exhibited high apparent viscosity and low structural stability, indicating strong internal flow resistance and poor shape retention. This behavior may limit filament stability and deposition accuracy during extrusion.

The rheological properties of the system were significantly affected by the addition of surimi, moisture, and butter. As shown in Figure 7(A-1,A-2), with increasing surimi and moisture content, the apparent viscosity of the system first decreased and then showed a gradual increase. This behavior can be attributed to the disruption and subsequent re-establishment of the internal network structure. At lower addition levels, surimi and moisture act as diluting components that weaken the gluten-based network continuity, resulting in reduced structural resistance. At higher surimi concentrations, protein–protein interactions become more dominant, promoting the formation of a more compact composite network with starch and residual gluten, thereby increasing system viscosity [39]. In addition, butter addition led to an increase in apparent viscosity (Figure 7(A-3)), which may be attributed to the formation of a dispersed fat phase that interacts with the continuous protein–starch matrix, enhancing the apparent structural resistance of the system. The system therefore evolves from a relatively homogeneous starch–protein dispersion into a more complex multiphase structured system.

The composite modulus (G*) further reflects the overall viscoelastic strengthening of the system after formulation optimization (Figure 7B). The addition of surimi and butter increased G*, while moisture addition reduced it, indicating their respective roles in reinforcing or plasticizing the network structure. These changes in G* are consistent with the observed improvements in printing stability and shape retention. Therefore, under the combined effects of protein, polysaccharide, and lipid interactions, the system transitions from a simple starch-based batter to a structured composite gel system with enhanced overall viscoelastic resistance, which is the fundamental reason for its improved 3D printing performance.

#### 3.4.4. Microstructure

**Figure 8:**
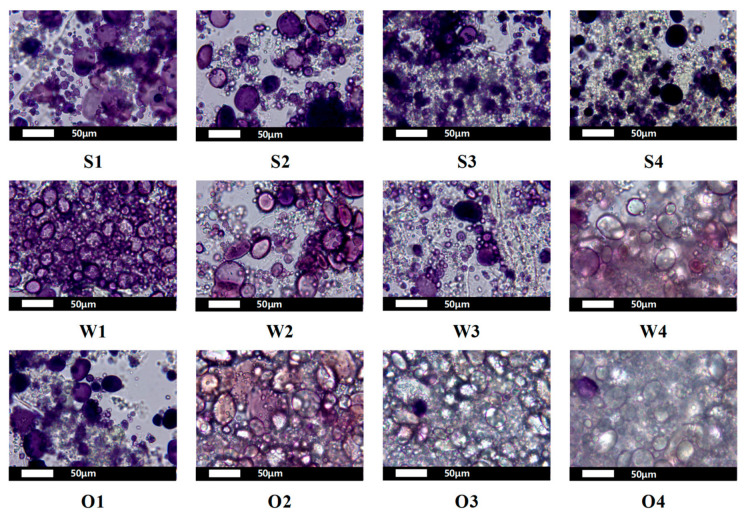
The effect of ingredient composition on the microstructure of surimi–flour composite batter. (S—surimi addition; W—moisture addition; O—butter addition). Data are presented as mean ± SD (*n* = 3).

The evolution of the starch microstructure can be effectively observed via the iodine–potassium iodide staining method. At room temperature, flour is stably stained dark blue to purple-black by the iodine–potassium iodide solution, while proteins show no chromogenic reaction, thus enabling the clear differentiation of their distribution states in the mixed system [40,41]. As shown in Figure 8, pure flour tends to aggregate. The incorporation of surimi into the flour batter results in its coating on the surface of flour particles, which facilitates the dispersion of flour particles. After moisture addition, the flour particles are further dispersed, which might be attributed to the unfolding of myofibrillar proteins due to sufficient hydration, which gradually coat the surface of flour particles to form an interfacial film. This change reveals the key role of moisture in promoting the homogeneity of protein–starch mixing and reducing the cohesive strength of the system. The subsequent addition of butter on this basis further remodels the internal structure of the system. Fat particles were distributed within the preliminarily formed protein–starch interfacial regions. This not only enhances the continuity of the interfacial structure but also effectively reduces the frictional resistance and van der Waals forces between starch-protein aggregates [42].

#### 3.4.5. CLSM Analysis

**Figure 9:**
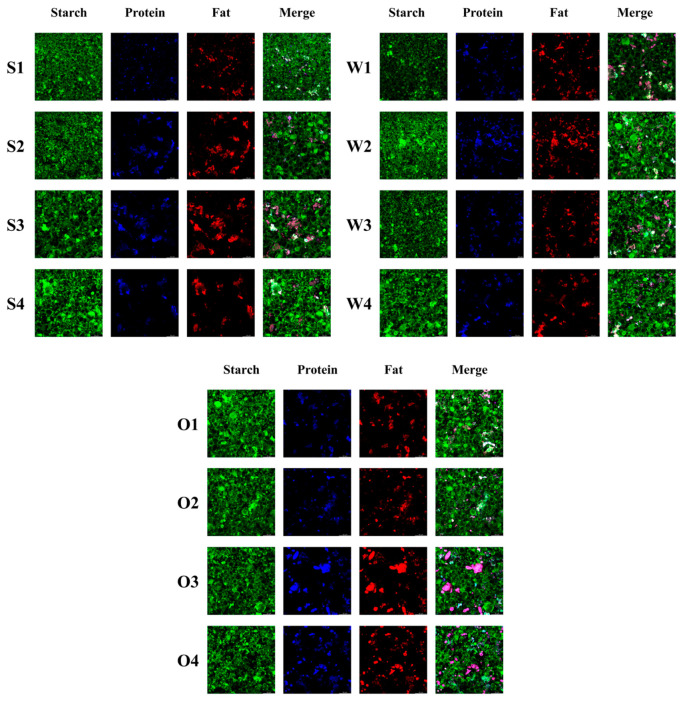
CLSM micrographs of surimi–flour composite batter with different formulations (S—surimi addition; W—moisture addition; O—butter addition). Columns from left to right represent starch (ConA, green), protein (FITC, blue), fat (Nile Red, red), and merged fluorescence images. Scale bars = 50 μm.

The microstructure of surimi–flour composite batters was further characterized by CLSM to investigate the spatial distribution of protein (green), starch-associated regions (blue), and lipid components derived from butter (red) within the composite systems (Figure 9). In the pure flour system (S1), starch-associated regions showed a relatively aggregated distribution, while protein signals were weak and discontinuous. With increasing surimi addition (S2–S4), both the intensity and distribution of protein signals increased, accompanied by enhanced overlap between protein and starch-associated regions in merged images. This suggests that surimi incorporation altered the spatial distribution of protein and starch components, with increased overlap between these regions, indicating improved structural organization of the composite matrix. These observations were consistent with the iodine-staining results. For moisture variation, increasing water content from 62% (W1) to 68% (W3) promoted a more uniform distribution of starch-associated regions and enhanced the continuity of protein regions, suggesting enhanced hydration and a more uniform distribution of starch and protein components. However, further increasing moisture to 71% (W4) resulted in a less compact microstructure, indicating that excessive water addition may weaken the structural organization of the composite matrix. These observations correspond well with the WHC and LF-NMR results. Regarding butter addition, moderate butter levels (O2–O3) resulted in a relatively uniform distribution of red fluorescent lipid droplets within the starch–protein matrix, with droplets mainly distributed in regions surrounding starch and protein domains. This suggests that an appropriate amount of butter may contribute to the formation of a more continuous composite structure. In contrast, excessive butter addition (O4) caused the formation of larger lipid aggregates and reduced the continuity of the starch–protein network, which may be associated with the decreased water-holding capacity observed previously.

Overall, CLSM observations revealed changes in the spatial organization of the composite batter, including reduced aggregation of starch-associated regions after surimi incorporation and altered lipid distribution following butter addition. These microstructural observations were consistent with the physicochemical variations discussed in the preceding sections.

#### 3.4.6. Particle Size

**Figure 10:**
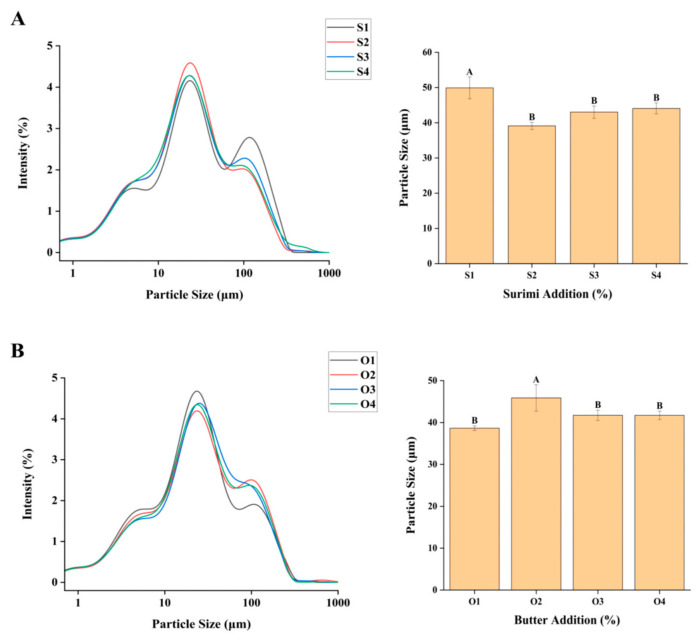
The effect of ingredient composition on the size distribution of surimi–flour composite batter. (**A**) Surimi addition; (**B**) butter addition. Different letters of the same parameter indicate significant differences between the average values (*p* < 0.05). Data are presented as mean ± SD (*n* = 3).

To verify the above analysis of the causes for microstructure changes, the particle size of the printing batter was determined using a laser particle size analyzer in this study. As shown in Figure 10, the particle size of the pure flour batter was 49.91 ± 0.02 μm. The incorporation of surimi resulted in a significant decrease in the particle size of the system (*p* < 0.05), and the subsequent addition of 3% butter caused a slight increase in the particle size of the batter, although the value remained lower than that of the pure flour batter. This indicates that the addition of surimi may promote a more uniform dispersion state of the composite system, reducing particle aggregation to a certain extent. The slight increase induced by butter addition may be attributed to the presence of dispersed lipid droplets within the continuous matrix, which slightly modifies the particle size distribution without disrupting the overall dispersion trend. Overall, these results suggest that formulation modification alters the dispersion state of the system, which is consistent with the observed rheological behavior and printing performance. It should be noted that the 1:1000 dilution used for laser diffraction measurement may affect the absolute particle size values by weakening original intermolecular interactions. Therefore, the results should be interpreted in terms of relative differences among samples rather than absolute microstructural characteristics.

#### 3.4.7. Thermal Denaturation Properties

**Figure 11:**
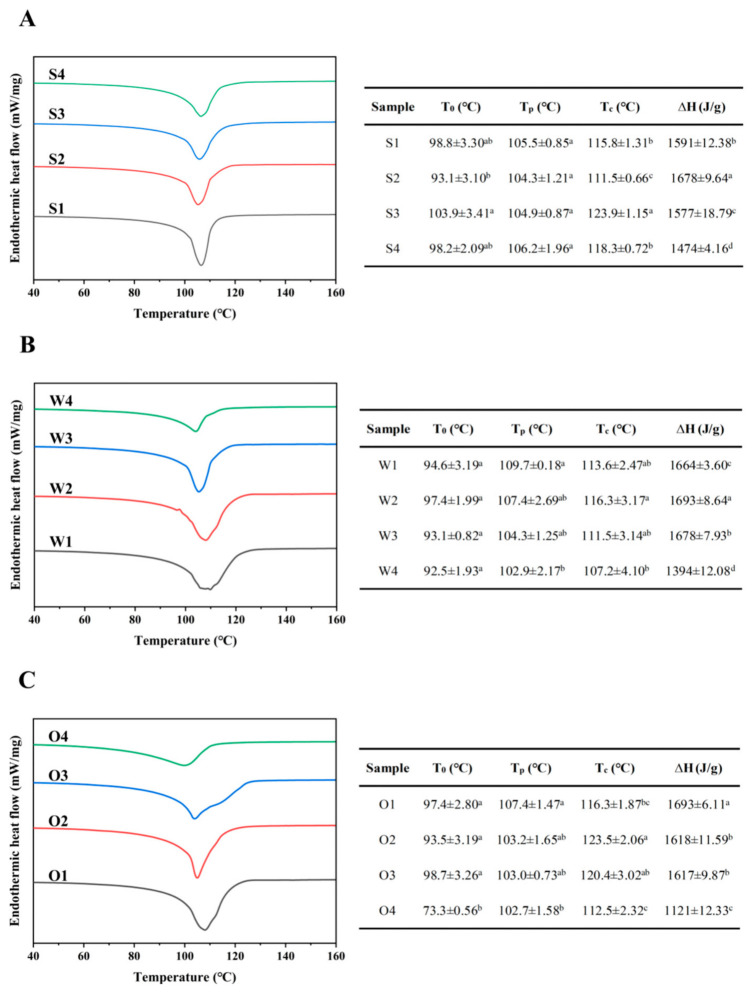
DSC thermograms of surimi–flour composite batters with different formulations. (**A**) surimi addition; (**B**) moisture addition; (**C**) butter addition. Different letters of the same parameter indicate significant differences between the average values (*p* < 0.05). Data are presented as mean ± SD (*n* = 3).

To investigate the effects of surimi, moisture and butter on the thermal properties of the printing batter during heating, differential scanning calorimetry (DSC) was employed for analysis in this study. The reported ΔH values represent the apparent thermal enthalpy of the entire wet composite batter (wet basis), as calculated automatically by the DSC software, rather than the gelatinization enthalpy of purified starch. As shown in Figure 11, all DSC curves exhibited a single endothermic peak, indicating that the mixture of surimi, polysaccharide and lipid did not introduce new crystal forms, and only affected the thermal transition behavior through intermolecular interactions. Further analysis of the thermodynamic parameters revealed that food ingredients exerted a significant regulatory effect on the thermodynamic properties of the flour batter. The incorporation of surimi into the flour batter increased the onset temperature (T_0_) and conclusion temperature (T_c_) of the system, while decreasing the enthalpy change (ΔH). This indicates that proteins in surimi coated starch granules and delayed the starch gelatinization process during heating. This trend was consistent with previous findings on cod surimi–wheat flour mixed systems [43], which reported that an increase in surimi addition significantly raised the thermal denaturation temperature of the system, confirming the universality of the inhibitory effect of surimi protein on starch gelatinization.

Subsequent moisture addition to the surimi–flour batter system led to increases in both T_C_ and ΔH, demonstrating that sufficient moisture promoted the complete gelatinization and crystal melting of starch. However, the gelatinization completion time was delayed due to the increased heat capacity of the system. The addition of butter resulted in a decrease in peak temperature (T_P_), an increase in T_C_ and a reduction in ΔH, reflecting that although lipids in butter accelerated the initial gelatinization response, they also delayed the late-stage gelatinization process. These changes suggest that butter modulates the thermal transition behavior of the composite batter, which may influence the thermal processing characteristics of printed constructs.

## 4. Conclusions

This study focused on the development and optimization of a 3D-printable surimi–flour composite batter, systematically investigating the effects of printing parameters and batter formulation on printing fidelity, shape retention, and extrusion performance, and further elucidating the underlying physicochemical mechanisms. The results demonstrated that batter flow rate (50 mm/s), mixing time (5 min), and syringe barrel volume (100 mL) were the key processing parameters for achieving continuous extrusion and stable deposition. In terms of batter formulation, the composite system with cake flour as the matrix and the incorporation of surimi, water, and butter significantly improved the printability of the batter. Further physicochemical analysis revealed that surimi addition promoted flour particle dispersion and facilitated the transformation of bound water into immobilized water. Appropriate water addition improved hydration and regulated fluidity and water-holding capacity of the batter. Butter incorporation contributed to favorable rheological characteristics, including shear-thinning behavior and viscoelastic properties. Studies on microstructure and thermal behavior indicated that the addition of these ingredients increased intermolecular interactions within the system and effectively delayed the starch gelatinization process.

In summary, high-fidelity 3D printing of the surimi–flour composite batter was successfully achieved through systematic optimization of printing parameters and batter formulation. The developed surimi–flour–butter composite system exhibited stable printability and improved printing performance, demonstrating its potential as a printable food ink for extrusion-based 3D food printing applications. The present study was conducted at a laboratory scale; therefore, its scalability and industrial feasibility still require further verification. In addition, formulation optimization was based on single-factor experiments, and the interactions among components were not quantitatively characterized. It should also be noted that this study focused on the printability of the raw batter formulation; the quality attributes of cooked constructs, including texture, color, and sensory properties, were not evaluated and remain to be investigated in future work. Future studies should further focus on process scale-up, system stability, and comprehensive optimization of formulation and processing parameters to support potential industrial applications.

## Figures and Tables

**Table 1:** Formulations of surimi–flour composite batter.

Code	Surimi (g)	Cake Flour (g)	Water (g)	Butter (g)
Surimi addition				
S1 (0%)	0	260	540	0
S2 (15%)	39	221	540	0
S3 (25%)	65	195	540	0
S4 (35%)	91	169	540	0
Water addition (based on S2)				
W1 (62%)	39	221	432	0
W2 (65%)	39	221	486	0
W3 (68%)	39	221	540	0
W4 (71%)	39	221	594	0
Butter addition (based on S2 + W2)				
O1 (0%)	39	221	486	0
O2 (3%)	39	221	486	22
O3 (6%)	39	221	486	44
O4 (9%)	39	221	486	67

Note: surimi addition = W(surimi)/W(surimi + cake flour); water addition = W(water)/W(surimi + cake flour + water); butter addition = W(butter)/W(surimi + cake flour + water + butter).

## Data Availability

The original contributions presented in this study are included in the article. Further inquiries can be directed to the corresponding author.
